# Constitutive Expression of TNF-Related Activation-Induced Cytokine (TRANCE)/Receptor Activating NF-κB Ligand (RANK)-L by Rat Plasmacytoid Dendritic Cells

**DOI:** 10.1371/journal.pone.0033713

**Published:** 2012-03-13

**Authors:** Thomas Anjubault, Jérôme Martin, François-Xavier Hubert, Camille Chauvin, Dominique Heymann, Régis Josien

**Affiliations:** 1 INSERM UMR 1064, Nantes, France; 2 CHU Nantes, Institut de Transplantation Urologie Néphrologie (ITUN), Nantes, France; 3 CHU Nantes, Laboratoire d'Immunologie, Nantes, France; 4 INSERM UMR 791, Nantes, France; 5 Université de Nantes, Faculté de Médecine, Nantes, France; Universität Würzburg, Germany

## Abstract

Plasmacytoid dendritic cells (pDCs) are a subset of DCs whose major function relies on their capacity to produce large amount of type I IFN upon stimulation via TLR 7 and 9. This function is evolutionary conserved and place pDC in critical position in the innate immune response to virus. Here we show that rat pDC constitutively express TNF-related activation-induced cytokine (TRANCE) also known as Receptor-activating NF-κB ligand (RANKL). TRANCE/RANKL is a member of the TNF superfamily which plays a central role in osteoclastogenesis through its interaction with its receptor RANK. TRANCE/RANK interaction are also involved in lymphoid organogenesis as well as T cell/DC cross talk. Unlike conventional DC, rat CD4^high^ pDC were shown to constitutively express TRANCE/RANKL both at the mRNA and the surface protein level. TRANCE/RANKL was also induced on the CD4^low^ subsets of pDC following activation by CpG. The secreted form of TRANCE/RANKL was also produced by rat pDC. Of note, levels of mRNA, surface and secreted TRANCE/RANKL expression were similar to that observed for activated T cells. TRANCE/RANKL expression was found on pDC in all lymphoid organs as well blood and BM with a maximum expression in mesenteric lymph nodes. Despite this TRANCE/RANKL expression, we were unable to demonstrate in vitro osteoclastogenesis activity for rat pDC. Taken together, these data identifies pDC as novel source of TRANCE/RANKL in the immune system.

## Introduction

Dendritic cells (DC), as antigen presenting cells (APC), play a key role in the induction of adaptive immunity [1]. Several DC subsets with specific phenotype and function have been described in human and mouse model [2], [3]. The two main DC populations described are conventional DC (cDC) and plasmacytoid DC (pDC). pDC have been first described as plasmacytoid monocytes and plasmacytoid T cells [4]). They were finally described as DC in the 1990s [5], and were shown to be the natural IFN-producing cells [6]. Indeed, pDC produce enormous amounts of type 1 interferon (IFN) upon virus recognition [7] which is mediated by TLR7 and 9, two TLR strongly expressed in pDC [8]. pDC subsets with different phenotype and function have been recently described. In human, CD2^+^ and CD2^−^ pDC have been described with CD2^+^ pDC exhibiting an in vitro cytotoxic activity and expression of lysozyme [9]. CD123^high^ and ^low^ pDC have also been described in patients with multiple sclerosis in which the predominance of one or the other subset is correlated with the gravity of the disease [10]. In mouse, CD4^+^ and CD4^−^ pDC have been identified with CD4^−^ pDC being the major source pDC subset that migrate into lymph nodes in response to infection [11]. A subset of CCR9^+^ pDC was recently shown to exhibit tolerogenic functions [12].

TRANCE is a member of the TNF superfamily, also called receptor activator of NF-κB ligand (RANKL), osteoprotegerin ligand (OPGL), osteoclast differentiation factor (ODF), TNFSF11 and CD254 [13]. TRANCE binds to the receptor activator of NF-κB (RANK) also known as TRANCE-receptor, and to the decoy receptor osteoprotegerin (OPG). TRANCE is expressed mainly on osteoblasts and stromal [14], [15] inducing maturation and activation of osteoclast (OC) precursor in fully functional OC able to degrade bone matrix. Stromal cells, activated T and B cells were also shown to express TRANCE [16], [17], [18]. Besides playing key role in osteoclastogenesis [13], TRANCE-RANK interaction promotes mature DCs survival and cytokine secretion in DC [17]. In vivo, TRANCE-RANK interaction was shown to play an important role in CD40L-independant CD4^+^ T cell response to virus [19] and to be required for lymph node organogenesis [20]. Indeed, mice deficient for TRANCE gene lacks all the lymph nodes. They also exhibit severe osteopetrosis, a consequence of their lack of osteoclasts. In fact, TRANCE plays a key role in linking bone physiology and immune system.

We report, herein, that rat pDC constitutively expressed TRANCE mRNA and confirmed the expression of both membrane and soluble form of the protein.

## Results and Discussion

### Constitutive TRANCE mRNA expression by rat pDC

**Figure 1:**
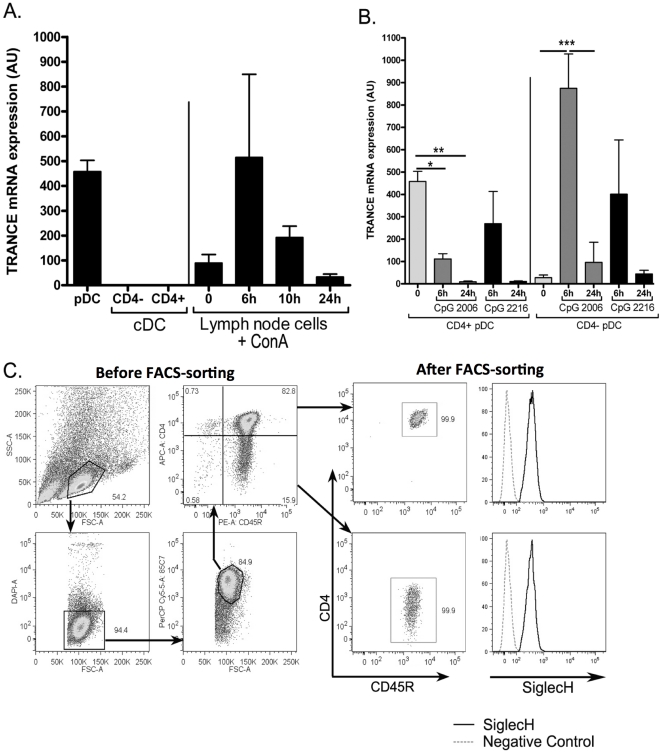
TRANCE mRNA expression on DC and pDC subsets. **A.** The expression of TRANCE mRNA was assessed in total mRNA prepared from FACS-sorted spleen cDCs and pDCs (n = 3) and from lymph node cells (n = 3) before and after activation with ConA. **B.** Gating strategy for sorting pDC subsets. After positive selection of 85C7^+^ cell, cells were stained with anti-CD45R and anti-CD4 mAbs, then sorted as 85C7^+^CD45R^+^CD4^high^ and CD4^low^ cells (dot plots, before FACS-sorting). Purity was routinely >99.5% for both population (Dot plots, after FACS-sorting). Sorted pDC subsets were also stained with anti-Siglec-H Ab (histrograms, after FACS-sorting). **C.** The expression of TRANCE mRNA was assessed in CD4^+^ and CD4^−^ subsets of pDCs at resting state and after maturation in presence of either CpG A or B. The results are expressed as a ratio of TRANCE to HPRT mRNA expression and represent the mean ± SD of three independent experiments. *, p<0,05; **, p<0,01; ***, p<0.001.

We previously described three DC subsets in rat spleen [21]. Conventional DCs (cDCs) expressed CD103 (clone OX62) and can be separated into CD4^+^SIRPα^+^ and CD4^−^ SIRPα^−^ cDCs [22]. We described rat plasmacytoid DC as CD103^−^ CD11b^−^ CD45^+^ and CD4^high^ cells [23]. We sought to identify genes specifically expressed in these spleen DC subsets and global gene expression was thus assessed in freshly extracted as well as TLR9-stimulated CD4^+^ and CD4^−^ cDC, and pDC using rat specific gene arrays. Among the genes specifically expressed in resting pDC, we identified TRANCE. We confirmed these data using real time QPCR (Fig. 1A) and observed that the level of TRANCE mRNA expression in resting pDC was similar to that observed at the peak of expression in activated T cells which are known to strongly expressed TRANCE [24]. In addition, TRANCE mRNA was not expressed in CD4^+^ and CD4^−^ cDCs, at the resting state.

Using a newly generated monoclonal antibody against rat pDC (clone 85C7), we recently identified two pDC subsets based on the expression of CD4 (Fig. 1B, left dot-plots) (Anjubault et al, unpublished results), as recently shown in mice [11]. In the spleen, the majority of pDC expressed high levels of CD4 [23], with CD4^low^ pDC representing 10–20% of total 85C7^+^ cells (Fig. 1B). After sorting of CD4^high^ and CD4^low^ cells, we observed that both of these subsets share features of pDC usually described (same morphology, expression of TLR7 and 9, type I IFN production) and expressed similarly siglec-H (Fig. 1B, right histograms) a pDC specific marker identified in mice [25]. However, as shown in figure 1C, TRANCE expression appeared to be differentially regulated in these subsets. A high level of TRANCE mRNA expression by resting CD4^high^ pDC was confirmed and this expression was rapidly down-regulated after type B CpG-induced maturation. Similar results were obtained after stimulation by CD40L or influenza (data not shown). Type A CpG appeared to maintain TRANCE expression during the first 6 hours but induced a dramatic down regulation at 24 h. In CD4^low^ pDC, a very low level of TRANCE mRNA was detected at resting state (Fig. 1C) but TLR9 stimulation induced a strong upregulation of TRANCE at 6 h followed by a down regulation at 24 h. At 6 h, the levels of TRANCE expression in CD4^low^ pDC were 2-fold higher than in resting CD4^high^ pDC. We also assessed TRANCE expression in TLR3 (polyI:C), TLR4 (LPS) and TLR9 (CpG B)-activated spleen CD4^+^ and CD4^−^ cDC subsets but did not observed any significant upregulation of TRANCE mRNA as compared to resting cells (data not shown).

The mRNA for TRANCE-receptor, also called RANK, was not expressed by DC at resting state but was strongly upregulated after maturation in cDC as we previously reported [17], and to a much lesser extent in pDC (data not shown). However, unlike on cDC, RANK protein was not detected on the surface of mature pDC (data not shown). A very similar expression profile was observed for the decoy receptor of TRANCE, OPG, yet with a higher expression at resting state for CD4^+^ cDC (data not shown).

### Rat pDC express membrane TRANCE protein

**Figure 2:**
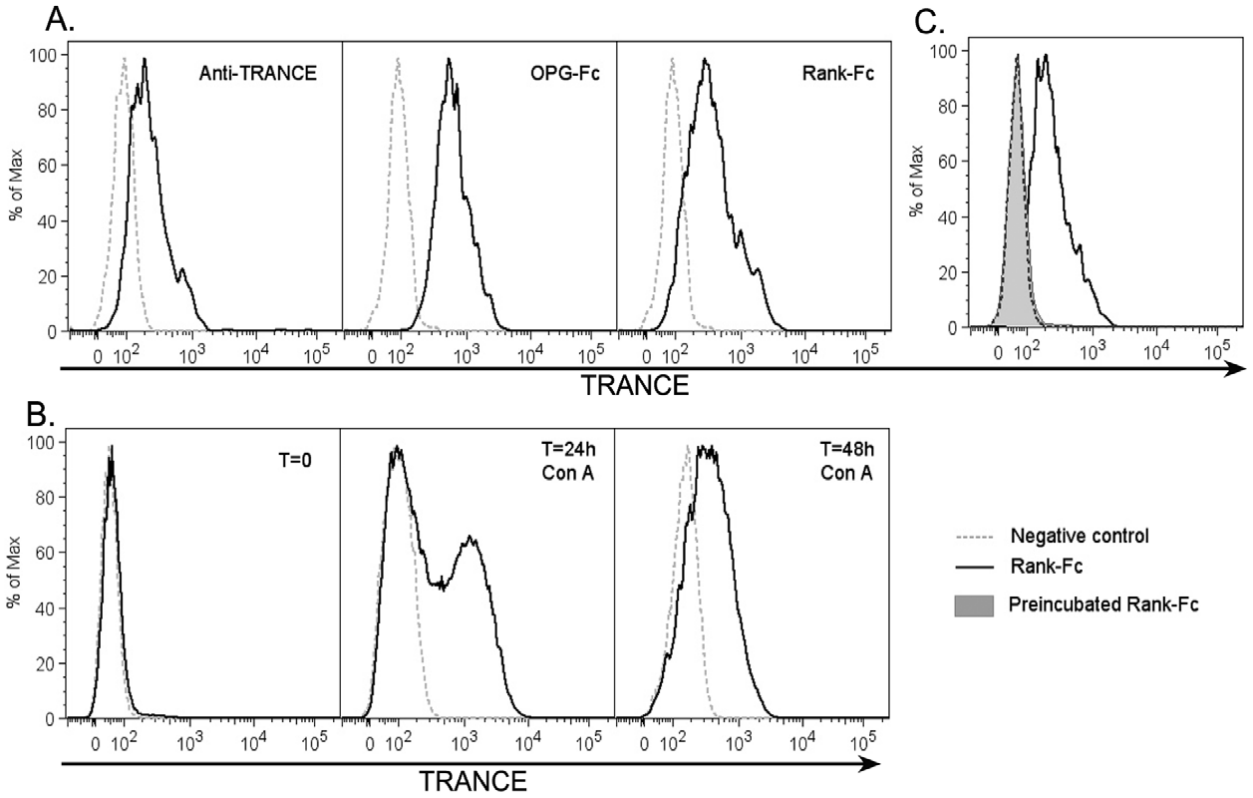
Surface TRANCE protein detection. **A.** Freshly FACS sorted CD4^high^ pDC were stained with a polyclonal antibody to TRANCE, OPG-Fc or RANK-Fc. **B.** Resting and Con-A-activated lymph node cells were stained with RANK-Fc and used as a positive control. **C.** RANK-Fc was preincubated with soluble TRANCE for 20 minutes at 4°C before staining pDC as in A.

**Figure 3:**
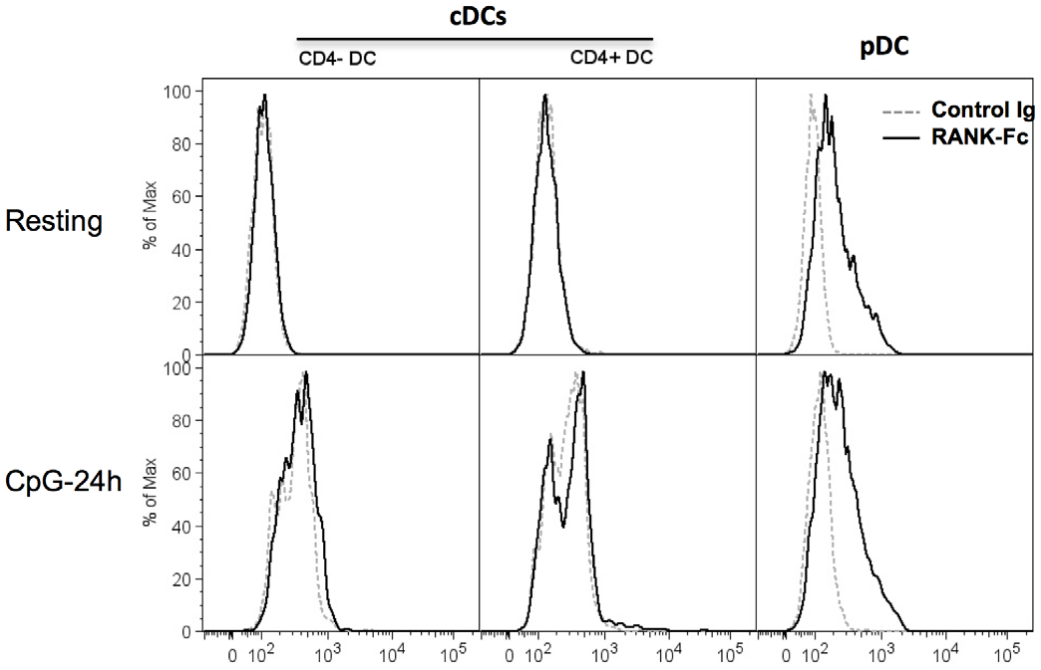
TRANCE protein expression on spleen DC subsets. Freshly isolated (upper histograms) or 24 h CpG B-stimulated (lower histrograms) CD4^+^ cDCs, CD4^−^ cDCs and CD4^high^ pDC or were stained with RANK-Fc (bold histrogram) or control Ig (dotted histogram).

TRANCE protein expression on pDC was assessed using a polyclonal Ab to mTRANCE, a fusion protein between murine RANK and a human Fc fragment (RANK-Fc), or OPG-Fc. We observe staining on CD4^high^ pDC with all these reagents (Fig. 2A). OPG-Fc gave the strongest signal but because OPG is known to also bind to TNF-related apoptosis inducing ligand (TRAIL) [26] and because pDC can express TRAIL under certain conditions [27], we decided to use Rank-Fc in the following experiments. The specificity of Rank-Fc staining was confirmed by blocking the fusion protein with soluble TRANCE as shown in figure 2B. As a positive control, we used, as we previously described [24], ConA-activated lymph node T cells which exhibited surface TRANCE expression after 24 h stimulation (Fig. 2C). Confirming the PCR data, we observed constitutive TRANCE expression on resting CD4^high^ pDC but not on CD4^+^ or CD4^−^ cDC (Fig. 3). This expression was maintained after 24 h of stimulation by CpG whereas it was not detected on TLR9 (Fig. 3) TLR3 or TLR4 (data not shown) activated CD4^+^ or CD4^−^ spleen cDC.

**Figure 4:**
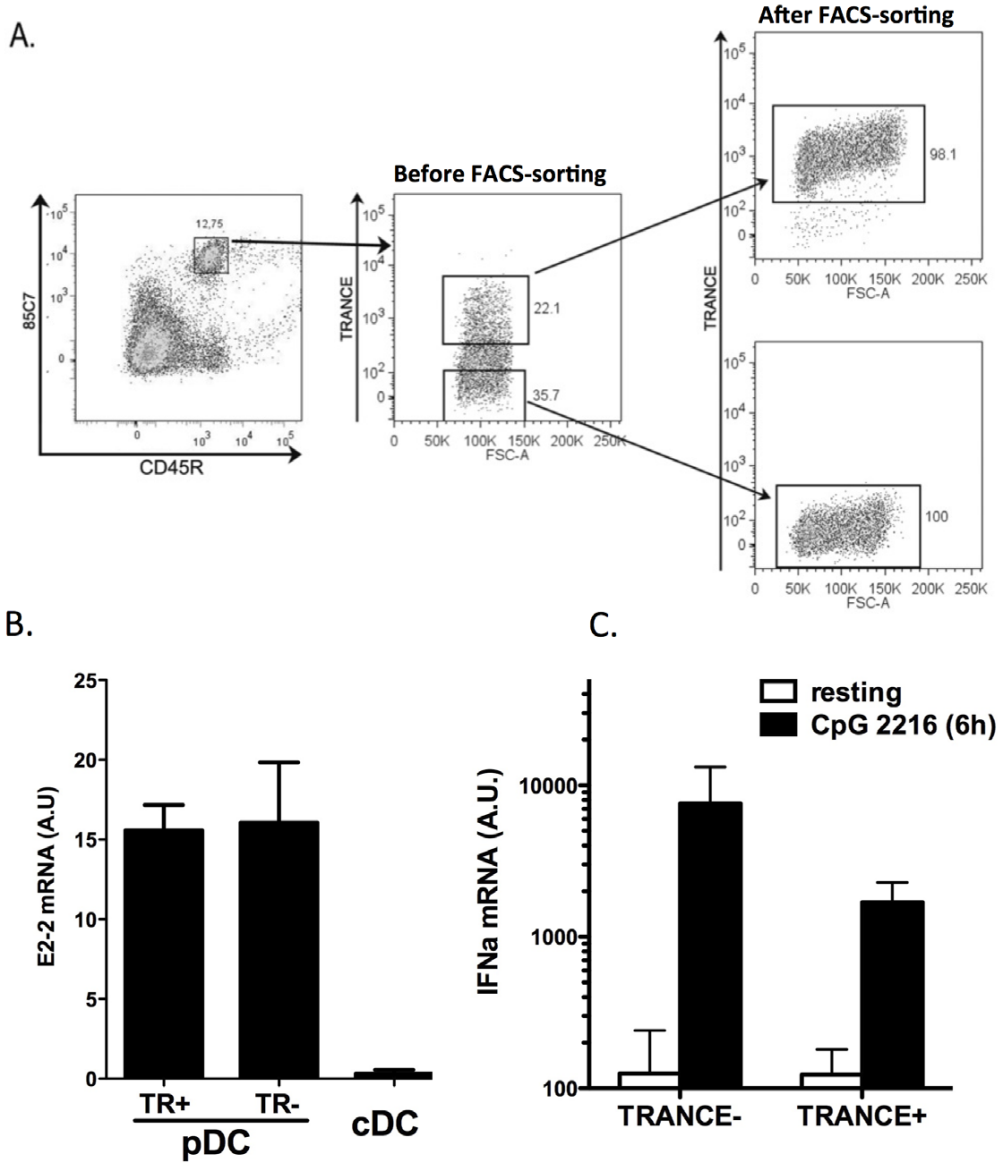
E2-2 and IFNα expression by TRANCE+ and − pDCs. **A.** Gating strategy for isolating TRANCE^+^ and ^−^ spleen pDC. After positive selection of 85C7^+^ cells (left dot plot), cells were stained with CD45R mAb and Rank-Fc. TRANCE^−^ and TRANCE^+^ 85C7^+^CD45R^+^ cells were sorted on a FACS Aria (middle dot plot). Purity was >98% for both population (right dot plots). **B.** E2-2 mRNA expression was assessed by Q-PCR in FACS sorted TRANCE+ (TR+) and – (TR−) population as well as in splenic cDCs (n = 2). **C.** The expression of IFNα mRNA was assessed by Q-PCR in FACS-sorted population (n = 3) as well as in 85C7^+^CD45R^+^ cells (total pDC), at resting state and after 6 h activation with CpG 2216 (5 µM). The results are expressed as a ratio of IFNα to HPRT mRNA expression and represent the mean ± SD of two or three independent experiments.

It was important to confirm that TRANCE-expressing 85C7^+^ CD45R^+^ cells have indeed pDC features. Therefore we sorted TRANCE^+^ and TRANCE^−^ 85C7^+^ CD45R^+^ cells and (Fig. 4A) compared E2-2 (a transcription factor required for pDC differentiation and function [28]) expression in these cells as well as IFNα expression after stimulation with type A CpG. As shown in figure 4B, both TRANCE^+^ and TRANCE^−^ 85C7^+^ CD45R^+^ cells similarly expressed high levels of E2-2 mRNA as compared to spleen cDCs. In the absence of available rat IFNα specific Ab, we assessed its expression by QPCR. As shown in figure 4C, we observed a strong induction of IFNα mRNA expression upon TLR9 triggering in both subsets, this induction being nevertheless stronger in TRANCE^−^ as compared to TRANCE^+^ 85C7^+^ CD45^+^ cells. Taken together, these data confirm that TRANCE expressing 85C7^+^ CD45R^+^ cells are pDC.

Because rat pDC express CD45R, a B cells marker, and because mouse and human B cells were described to express TRANCE after maturation [29], [30], we investigate TRANCE expression on resting and activated sorted rat B cells. In our hand, resting or activated B cells did not express TRANCE protein (data not shown). Previous studies have shown that TRANCE is constitutively expressed on a subset of CD4^+^CD3^−^ progenitor cells that are involved in induction of lymphoid tissues [31]. Although we could not completely exclude that such cells could contaminate our pDC suspensions, the extremely low frequency of lymphoid tissues inducer cells in adults lymphoid organs as compared to that of pDC exclude such a contamination accounted for the TRANCE expression we observed on pDC.

**Figure 5:**
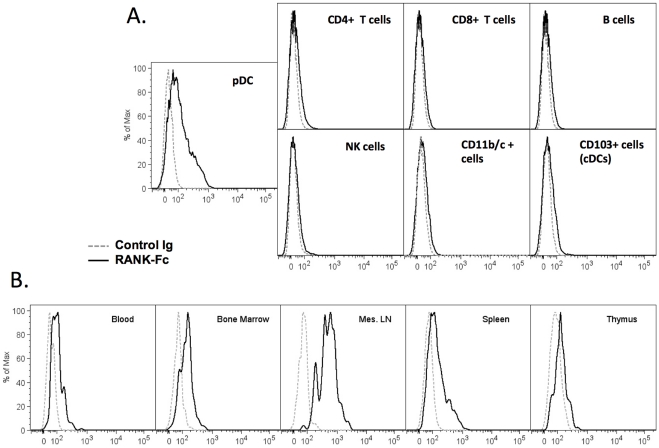
TRANCE expression on pDC in various lymphoid organs. **A.** Freshly prepared spleen cells were stained with various antibodies to identify CD4^+^ T cells (CD3^+^CD4^+^), CD8^+^ T cells (CD3^+^CD8^+^), B cells (CD45R^+^OX33^+^), NK cells (NKR-P1A ^high^CD3^−^), CD11b/c^+^ cells, cDC (CD103^+^), pDC (85C7^+^ CD4^+^) together with RANK-Fc (bold histogram) or the secondary Ab alone (dotted histogram). **B.** Cell suspensions from various lymphoid organs were stained with 85C7 and CD4 antibodies to identify CD4^high^ pDC and RANK-Fc (bold histogram) or the secondary Ab alone (dotted histogram). Similar results were obtained in 3 independent experiments for each panel.

We also assessed TRANCE expression on various immune cell subsets in rat spleen (CD4^+^ and CD8^+^ T cells, B cells, NK cells, CD11b/c^+^ cells, cDC). Unlike pDC, all these cells stained negative for TRANCE (Fig. 5A). When pDC were permeabilized, we could actually detect higher expression of TRANCE (data not shown) indicating strong intracellular expression. The difference between intracellular and membrane expression could be due to the production of the soluble form of TRANCE instead of the membrane form or could also be due to the cleavage of the membrane form by the metalloprotease TACE (ADAM17) which is known to release the membrane form of TRANCE into a soluble form [32]. Next we assessed TRANCE expression on pDC from different immune compartments (lymph node, thymus, bone marrow and blood) (Fig. 5B). In all organs analyzed, pDC were found to express TRANCE yet at different levels. A significant highest expression of TRANCE was found on pDC from mesenteric lymph nodes (mean (n = 3) of MFIs (SD) for TRANCE expression on pDC from: Blood: 92 (62); BM: 80 (26): MLN: 316 (168); Spleen:112 (9); Thymus: 55 (11)).

**Figure 6:**
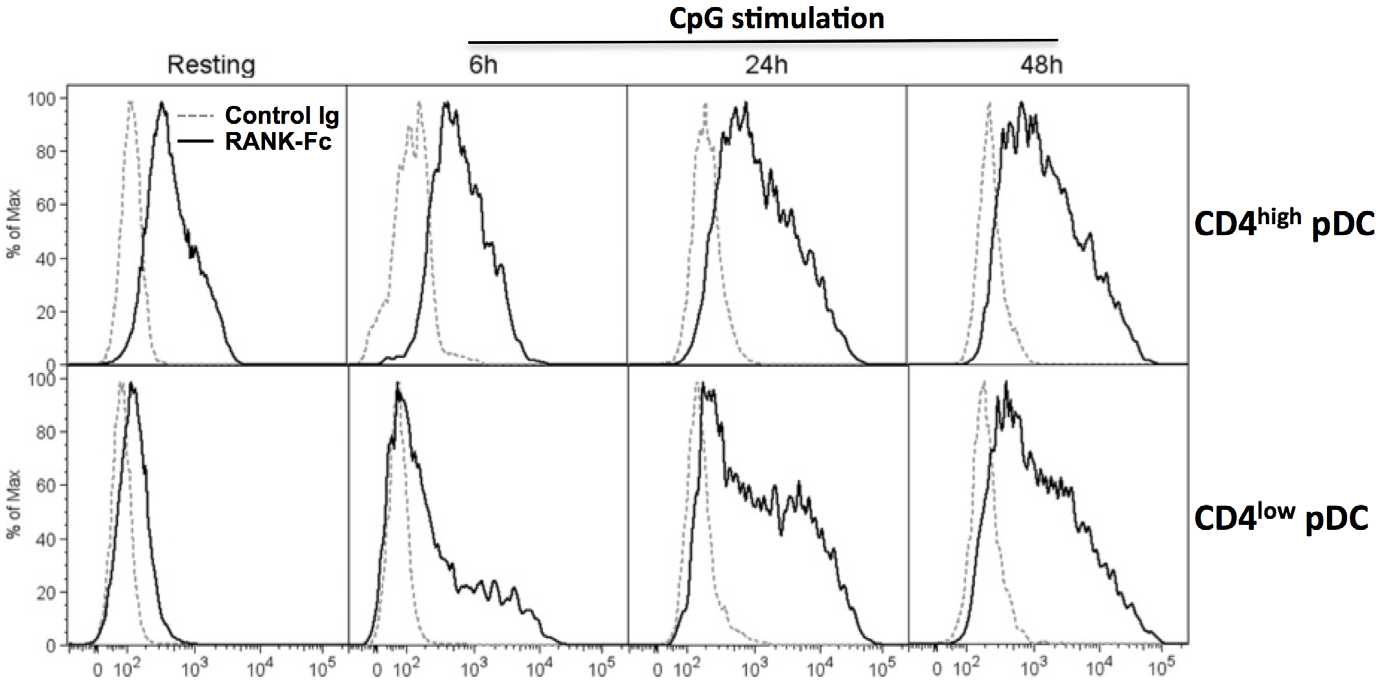
Kinetics of TRANCE protein expression on activated pDCs. FACS sorted CD4^high^ (upper panels) and CD4^low^ (lower panels) pDC were stimulated with CpG B and stained stained with RANK-Fc (bold lines) or the secondary Ab alone (thin lines) at 0 (resting), 6, 24 and 48 h after stimulation. Similar results were obtained in 3 independent experiments.

We compared the kinetics of TRANCE expression on purified CD4^high^ versus CD4^low^ pDC (Fig. 6) following TLR9 stimulation. As expected, TRANCE protein was expressed at very low levels on resting CD4^low^ pDC. Following activation by CpG, TRANCE expression was maintained on CD4^high^ pDC for at least 48 h whereas its expression was strongly induced on CD4^low^ pDC. It is interesting to note that CD4^low^ pDC are the predominant subset in bone marrow and blood (data not shown).

We failed to detect TRANCE expression in spleen pDC in mice either at the mRNA (Fig. S1) or the membrane or soluble protein levels (data not shown). In human, analysis of public data from gene array experiment (http://amazonia.transcriptome.eu/; http://www.immgen.org/index_content.html), indicated that resting human pDC did not express TRANCE mRNA. This was confirmed by RT-PCR analysis of blood DC subsets (data not shown) TRANCE being highly conserved between species and pDC also, it is surprising that TRANCE expression on pDC is restricted to rat model. It could be due to evolutionary modifications specific to rat phylogeny.

### Soluble TRANCE protein production by pDC

**Figure 7:**
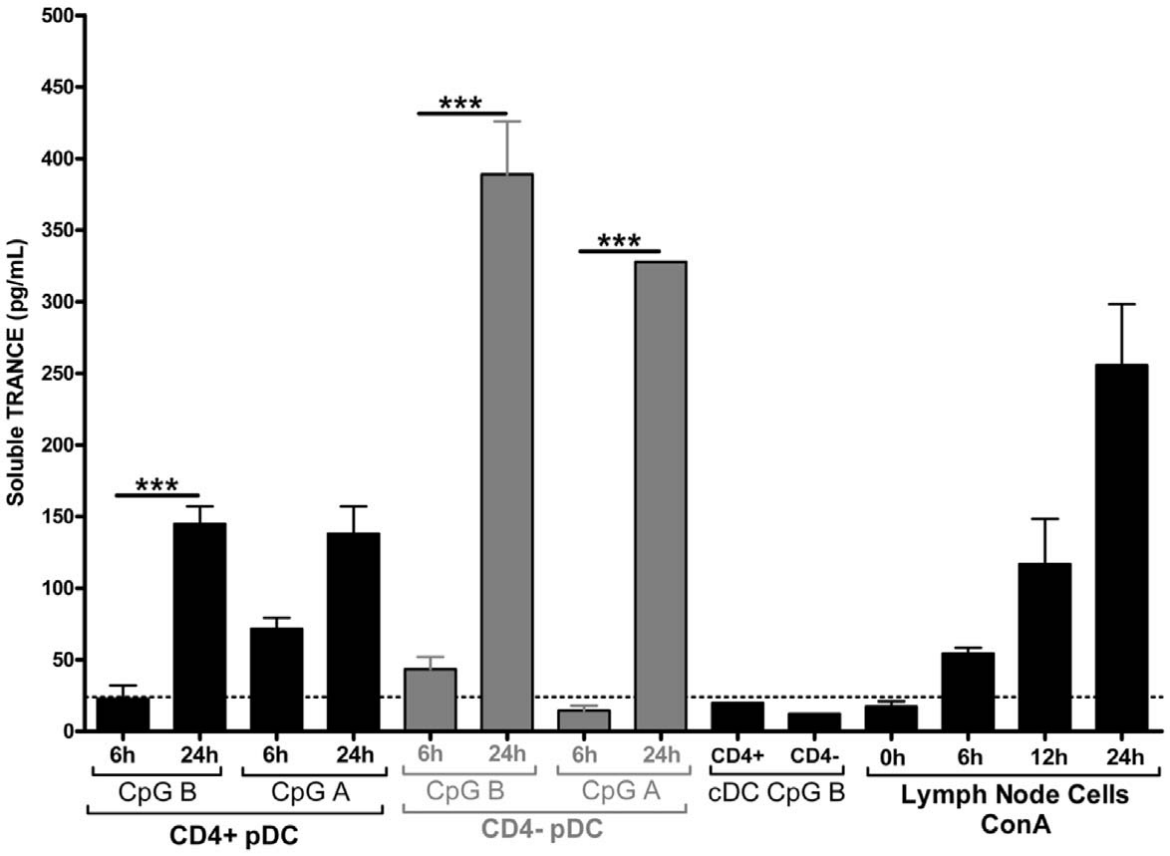
Soluble TRANCE production by pDCs. Freshly sorted splenic pDC (n = 4) and cDC (n = 3) were stimulated in complete RPMI with CpG B or A (5 µM). Lymph node cells were stimulated with ConA (5 µg/mL). Supernatants were collected at 6 and 24 h and soluble TRANCE was assessed by ELISA (R&D systems). The detection threshold of the ELISA was 24 pg/mL. ***, p<0.001.

In addition to its membrane bound form, TRANCE can also be produced as a soluble protein. Using an available ELISA for mouse TRANCE, we were able to detect rat TRANCE (Fig. 7). In supernatants from ConA-stimulated lymph node T cells which serve as positive control, we could detect an increased quantity of soluble TRANCE from 6 h to 48 h of culture. We did not detect significant levels of TRANCE in the supernatant of either CD4^+^ or CD4^−^ cDC. After 24 h stimulation with TLR 9 ligands, CD4^high^ pDC produce 150 pg/mL±12 pg/mL of soluble TRANCE. Consistent with their higher expression of TRANCE mRNA after stimulation, CD4^low^ pDC produce 2-fold more soluble TRANCE when they were cultured 24 h with a TLR9 ligand. Interestingly, these levels were higher than that produced by activated T cells. We could not detect soluble TRANCE in the supernatants of purified rat B cells cultured 24 h and 48 h with CpG A or B or LPS nor in the supernatants of CpG of LPS-activated cDCs subsets (data not shown).

### Lack of pDC-mediated osteoclastogenesis

**Figure 8:**
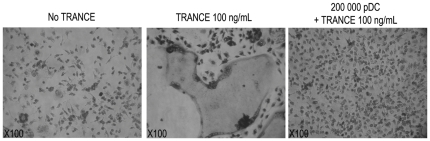
pDCs inhibits in vitro osteoclastogenesis. Bone marrow cells were differentiated in osteoclast and stained for their expression of tartrate-resistant acid phosphatase (TRAP) (Leukocyte Acid Phosphatase Assay kit; Sigma). Soluble TRANCE was used 100 ng/mL as a positive control for the induction of osteoclasts. When indicated, 2×10^5^ FACS sorted CD4^high^ pDC were added to bone marrow cells at day 0 in addition to TRANCE. pDCs were shown to inhibit TRANCE-mediated osteoclastogenesis in these conditions. These results are representative of 5 independent experiments.

We finally sought to assess whether rat pDC could induce osteoclastogenesis in vitro. Indeed, osteoclastogenesis mediated by TRANCE expressing T cells is already described [33]. We found that CD4^high^ and CD4^low^ pDC, after a short-term stimulation or 24 h with CpG 2006, were not able to promote osteoclastogenesis (data not shown) and surprisingly rather inhibit soluble TRANCE-mediated in vitro osteoclastogenesis (Fig. 8). Fixed cells also failed to induce osteoclastogenesis suggesting that soluble factors produced by pDC such as IFNα [34] which are known to inhibit osteoclastogenesis were not involved. Although the amount of soluble TRANCE produced by pDC is almost 1,000-fold lower than the quantity of exogenous TRANCE necessary to induce osteoclasts formation in vitro, activated T cells were shown to induce osteoclastogenesis in the same conditions. Therefore, these negatitve in vitro data do not exclude a potential role of pDC in regulating bone homeostasis in vivo and, moreover, pDC might cooperate with other cells such as osteoblastic stromal cells, osteoblasts or bone marrow stromal cells which, in response to IL-6 produced by activated pDC, can produce soluble TRANCE [35].

### Conclusion

In conclusion, our data indicate that rat CD4^high^ pDC constitutively expressed TRANCE both at the mRNA and the protein levels. Although TRANCE is expressed at very low levels on resting CD4^low^ pDC, it is strongly upregulated after TLR-mediated activation. Whether CD4^low^ and CD4^high^ pDC represent different developmental stage or different subsets of pDC remains to be determined. However, the fact that CD4^low^ pDC were mainly found in bone marrow and blood while CD4^high^ pDC were dominant in lymphoid organs suggest that CD4^low^ pDC could be the precursors of CD4^high^ pDC. This is consistant with the recent description of CD4^−^ and CD4^+^ pDC subsets in mice [11]. TRANCE was found to be expressed by rat pDC as a membrane bound and a soluble protein, the levels of which were similar of higher than that produced by activated T cells. Intriguingly this specific phenotype of pDC appears to be rat-specific as we so far did not find evidence of TRANCE expression on murine or human pDC. The functional role of TRANCE on pDC remains to be determined. Given the critical role of TRANCE in osteoclastogenesis, pDC could play in vivo, such as activated T cells, a role in regulating bone metabolism in rats, although in vitro, we failed to demonstrate that pDC could induce osteoclastogenesis. A recent report also indicate that Treg have an important pro-metastatic function through their expression of TRANCE in breast cancer [36]. Of note, pDC infiltration of breast tumor was found to correlate with an adverse outcome [37]. Whether pDC can express TRANCE in tumor remains to be determined. Recent studies indicate that pDC could enhance cDC function through CD40L expression [38], [39]. In the rat immune system, pDC could therefore through their expression of TRANCE act as survival and activating factor for cDC for enhancing specific immune responses [17].

## Materials and Methods

### Animals

Lewis rats, from 8 to 12 week old were obtained from the Centre d'Elevage Janvier (Le Genest St Isles, France). All animal experiments were performed under specific pathogen-free conditions in accordance with the European Union Guidelines. All animal studies were conducted according to the guidelines of the French Agriculture Ministry. The studies were approved by the Veterinary Departmental Services committee (# E.44011).

### Reagents

The phosphodiester oligonucleotide containing the CpG motif (CpG ODN) 2006 (tcgtcgttttgtcgttttgtcgtt) and 2216 (ggGGGACGATCGTCgggggg) were synthesized by Eurofins MWG Operon (Ebersberg, Germany). Monensine, brefeldin A and Concanavaline A were obtained from Sigma-Aldrich (Saint-louis, MI).

### Antibodies

The Rank-human Fc fusion molecule and the mTRANCE-human CD8 fusion molecule were kindly provided by Yongwon Choi (University of Pennsylvania, Philadelphia). CD4-Phycoerythrine (PE)-Cy7, and CD45R-PE monoclonal antibody were purchased from BD Biosciences (Le Pont de Claix, France). R7/3 (TCRαβ), OX8 (CD8), OX12 (Igκ), OX33 (CD45RA), OX35 (CD4), OX42 (CD11b/c) hybridomas were obtained from the European Collection of Cell Culture, and mAb were purified from supernatants followed or not by coupling to Alexa Fluor 647 and Alexa Fluor 488 (Invitrogen, Cergy Pontoise, France). The anti-human Fc-FITC monoclonal antibody was purchased from Jackson ImmunoResearch Laboratories Europe Ltd (Suffolk, UK).

### Dendritic cells sorting

#### Conventional DC

CD4^+^ and CD4^−^ cDC were isolated as previously described [22]. Briefly, after digestion in collagenase D (Roche Diagnostics, Meylan, France), low density spleen cells were selected on a 14.5% Nycodenz (Nycomed, Oslo, Norway) gradient. CD103^+^ cells were then selected using OX62-MACS microbeads (Miltenyi Biotec, Paris, France) and stained with CD103-Alexa Fluor 647 (Clone OX62) and CD4-PE mAbs. CD103^+^ CD4^+^ and CD103+ CD4^−^ cells were sorted using a FACS Aria (BD Biosciences).

#### Plasmacytoid DC

Spleens were perfused with 2 mg/mL collagenase D in RPMI 1640/1% FCS, chopped into small pieces and incubated 25 min at 37°C. EDTA (10 mM) was added, and the cell suspension was pipetted up and down for 5 min and filtered on 100 µm. Cells were washed in PBS/0.5 mM EDTA/2% FCS and mononuclear cells were isolated by centrifugation over Ficoll-Plaque Plus (Amersham, Les Ulis, France). T and partial B cell depletion was then performed by incubating cells with CD8 (OX8), Igκ (OX12), CD45RA (OX33), CD11b/c (OX42), TCRαβ (R7.3) and TCRγδ (V65) mAbs followed by anti-mouse IgG-coated magnetic beads (Invitrogen). After staining with biotinylated 85C7 mAb, cells were incubated with anti-biotin conjugated MACS Microbeads following the manufacturer's intructions (Miltenyi Biotec). Positive selection was performed on AutoMACS Separator (Miltenyi Biotec). Cells were then stained with. 85C7^+^ CD45R^+^ CD4^high^ and 85C7^+^ CD45R^+^ CD4low cells were sorted on a FACS Aria (BD biosciences). Purity was routinely >99%. When indicated, 85C7+ enriched cells were stained with Streptavidine-PerCp Cy5.5, CD45R-PE (HIS24) mAbs and RANK-Fc followed by FITC anti-human Fc Ab and sorted on a FACS Aria.

### Flow cytometry analysis

Cells were stained using PBS diluted Ab and washed with PBS/0.2% FCS/0.01% Azide. Intracellular staining was realised using the Fixation/Permeabilization Diluent (eBiosciences, San Diego, CA) and the Permeabilization Buffer (10×) (eBiosciences) according to manufacturer's instructions. Cells were then acquired on a BD FACS LSRII (BD biosiences) and results were analyzed using FlowJo software (Treestar, Ashland, OR)).

### Real-Time Quantitative PCR

Total RNA from 2×10^5^ to 2×10^6^ resting or stimulated DC subsets and lymph node cells was extracted using Trizol (Life Technologies, Paisley, U.K.) and synthesized to cDNA. Real-time quantitative PCR was performed with this cDNA using an Applied Biosystems GenAmp 7700 Sequence Detection System. TaqMan probes sequences used were: rTRANCE forward: CAGAATATCAGAAGACAGCACGC; rTRANCE reverse: AGCCACGAACCTTCCATCAT, resulting in a 225-bp PCR product in rat model; and rHPRT forward: CCTTGGTCAAGCAGTACAGCC; rHPRT reverse: TTCGCTGATGACACAAACATGA, resulting in a 188-bp PCR product; mTRANCE forward: CACACCTCACCATCAATGCTG; mTRANCE reverse: AGAATTGCCCGACCAGTTTT resulting in a 307-bp PCR product in mouse model; Total cDNA was amplified in a 25 µL reaction PCR mix constituted of 10 µL of diluted cDNA, 12.5 µL of TaqMan SybrGreen 2× PCR Master Mix (Applied Biosystems, Applera, Courtaboeuf, France) containing AmpliTaq Gold DNA polymerase, dNTPs with dUTP and optimized buffer components, and 0.8 µL of 20× TaqMan probes. The reaction started with a step of 2 min at 50°C and 10 min at 95°C followed by 40 cycles consisting of 15 s at 95°C and 1 min at 60°C. Relative expression was calculated using the −2^−ΔΔCt^ method and expressed in arbitrary units.

### ELISA test

The amount of soluble TRANCE in the supernatants of DC and lymph node cell cultures was measured using a mouse ELISA kit (R&D Systems, Minneapolis, MN) according to the manufacturer's instructions.

### Osteoclast Differentiation Assay

Tibias and femurs were harvested under sterile condition and washed with 75% alcohol. Bone Marrow (BM) was washed out with PBS/0.5 mM EDTA/2% FCS using disposable syringes. Cells were washed after red blood cell lysis, and suspended at 10^7^ cells/mL in MEM Alpha Medium (GIBCO, USA)/10% FCS in 6-well culture plates. During the 16 first hours of culture at 37°C, BM cells are in the presence of 5 ng/mL of Macrophage Colony Stimulating Factor (M-CSF) (Peprotech, Rocky Hill, NJ). After this step, non-adherent cells are washed and cultured for 3 more days with 30 ng/mL M-CSF and 1 ng/mL transforming growth factor (TGF-β)(R&D systems). Then adherent cells, after washing, are cultured in the presence of 30 ng/mL M-CSF and various conditions: With or without soluble TRANCE at 100 ng/mL, in the presence or not of pDC, resting or stimulated 3 hours with 5 µM CpG ODN 2006 and in the presence or not of lymph node cells stimulated 24 h with 5 µg/mL Concanavaline A. After three days, presence or absence of osteoclasts is revealed by tartrate-resistant acid phosphatase (TRAP) staining (Leukocyte Acid Phosphatase Assay kit; Sigma) [26].

### Statistical analysis

Statistical significance was evaluated using a two-way ANOVA test and a Bonferoni Post-test. p≤0.05 were considered significant.

## Supporting Information

Figure S1 **Spleen murine pDC do not express TRANCE mRNA.** Spleen CD8^+^ and CD8^−^ cDC subset, total pDC (mpDCA1^+^ B220^+^ cells) as well as CD4^+^ and CD4^−^ pDC were FACS sorted. TRANCE mRNA expression was assessed by Q-PCR in resting cells or after 6 h stimulation with type B CpG for CD4^+^ and CD4^−^ pDC subsets. As positive control, we used lymph node cells stimulated by Concanavalin A for 6 h ou 24 h. Histograms represent the mean+SD of TRANCE mRNA expression (arbitrary units) of 3 independent experiments.
(TIFF)
