# Supplementary figures and images for: Constitutive Expression of TNF-Related Activation-Induced Cytokine (TRANCE)/Receptor Activating NF-κB Ligand (RANK)-L by Rat Plasmacytoid Dendritic Cells
Source: PLoS One. 2012 Mar 13;7(3):e33713. doi: 10.1371/journal.pone.0033713 (PMC3302772; doi:10.1371/journal.pone.0033713)

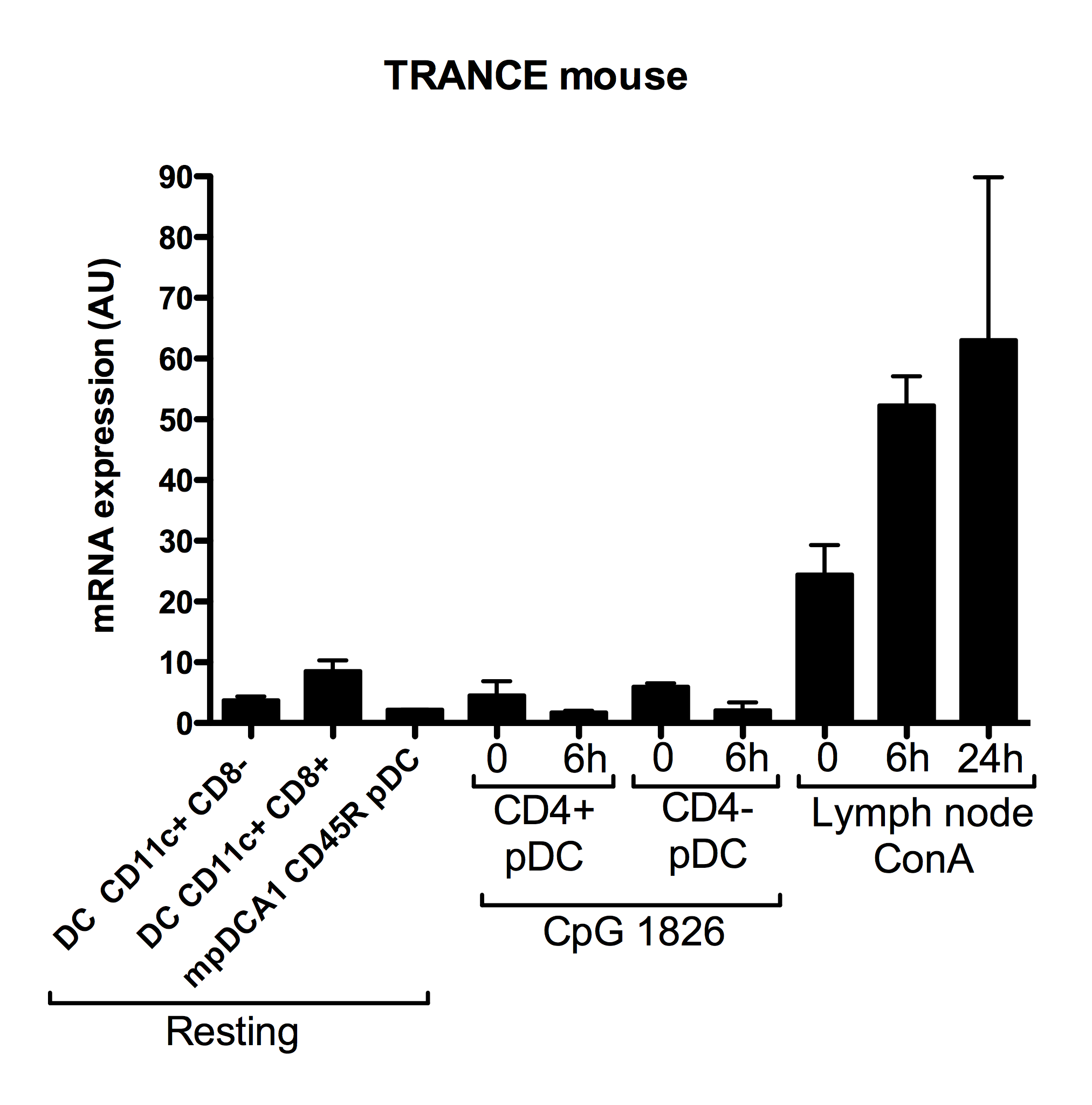

Supplement: Figure S1 — Spleen murine pDC do not express TRANCE mRNA. Spleen CD8+ and CD8− cDC subset, total pDC (mpDCA1+ B220+ cells) as well as CD4+ and CD4− pDC were FACS sorted. TRANCE mRNA expression was assessed by Q-PCR in resting cells or after 6 h stimulation with type B CpG for CD4+ and CD4− pDC subsets. As positive control, we used lymph node cells stimulated by Concanavalin A for 6 h ou 24 h. Histograms represent the mean+SD of TRANCE mRNA expression (arbitrary units) of 3 independent experiments. (TIFF) [file pone.0033713.s001.tiff]
